# Cellular and Molecular Mechanisms of Mesenchymal Stem Cell Actions

**DOI:** 10.1155/2017/2489041

**Published:** 2017-12-06

**Authors:** Bruno Christ, Marcella Franquesa, Mustapha Najimi, Luc J. W. van der Laan, Marc Hendrik Dahlke

**Affiliations:** ^1^University Hospital Leipzig, Leipzig, Germany; ^2^REMAR Group, Health Science Research Institute Germans Trias i Pujol (IGTP), Badalona, Spain; ^3^Université Catholique de Louvain, Brussels, Belgium; ^4^Erasmus MC-University Medical Center Rotterdam, Rotterdam, Netherlands; ^5^University Hospital Regensburg, Regensburg, Germany

The clinical interest in the therapeutic use of mesenchymal stromal/stem cells (MSC) is further increasing as their versatility in animal trial settings becomes more and more obvious. Indeed, screening the official review site for clinical trials (http://www.clinicaltrials.gov) using the search term “Mesenchymal Stem Cells” reveals 611 records (September, 2017). The large diversity of medical indications for treatment with MSC comprises hematological malignancies, diabetes type 1, neurologic diseases, joint and bone diseases, organ transplantation, or liver diseases, just to mention a small selection. This indicates the big potential of MSC to act beneficially both on chronic and acute diseases of either local or systemic origin. However, albeit we are only starting to understand the mode of action of MSC in certain disease conditions, there is still hesitation on the safety, upscale feasibility and, effectiveness of clinical MSC application.

MSC from different tissues and organs feature rather similar phenotypic characteristics when put in culture. These include the capacity of plastic adherence, multiple differentiation potential, and surface marker profiles, which comprise the minimal definition criteria for MSC [1]. But gaining knowledge of molecular signatures by global gene expression analyses currently reveals that heterogeneity exists between different MSC populations depending on their origin, isolation and propagation procedures, and on their status of differentiation [2–5]. The actions mediated by MSC may comprise two principally different mechanisms. The one is based on the functional integration of differentiated MSC into diseased host tissue after transplantation as has been shown for liver regeneration after partial hepatectomy or toxic injury [6, 7]. The second mechanism comprises paracrine or cellular support of self-restoration of the diseased tissue or organ [8, 9]. The impact of MSC on the regulation of both the innate and the acquired immune system was intensively investigated and sparked the application in the setting of organ transplantation, where immunosuppression of alloreactivity is essential to prevent rejection [10, 11]. It is mainly the involvement of key molecules like prostaglandin E2 (PGE2), indoleamine 2,3-dioxygenase, cytokines, and other growth factors, which act on cells of the immune system to activate or modulate their activity state and thus to impact on the immune status of the organ or organism as a whole.

Knowledge on the cellular targets of MSC actions is emerging. Yet, gain of knowledge still remains limited. Especially, effects on cell cycle and metabolism remain elusive which however are essential to predict potential adverse effects in the treatment of tumorigenic diseases like hepatitis [12] or diseases associated with the metabolic syndrome like diabetes type 2. Facing the pleiotropic properties of MSC like modulation of immune responses and alleviation of inflammation and tissue damage, as well as stimulation of tissue regeneration, it will be the goal of future efforts using relevant cell or animal model systems to unequivocally elucidate the molecular and cellular impact of a defined MSC population on a specified disease environment before their clinical application. In this special issue of Stem Cells International on the cellular and molecular mechanisms of MSC actions (CMMM), we provide a collection of work stepping towards this goal in order to complement gaps of knowledge before unequivocal use of MSC in clinical settings

*Bruno Christ* *Marcella Franquesa* *Mustapha Najimi* *Luc J. W. van der Laan* *Marc Hendrik Dahlke*
